# 2,2′-Hexamethyl­enedi-1,3-benzothia­zole

**DOI:** 10.1107/S1600536809000610

**Published:** 2009-01-14

**Authors:** Guo-wei Wang, Ling-hua Zhuang, Jin-tang Wang

**Affiliations:** a Department of Light Chemical Engineering, College of Science, Nanjing University of Technology, Nanjing 210009, People’s Republic of China; b Department of Applied Chemistry, College of Science, Nanjing University of Technology, Nanjing 210009, People’s Republic of China

## Abstract

The title compound, C_20_H_20_N_2_S_2_, was prepared by the reaction of suberic acid and 2-amino­thio­phenol under microwave irradiation. The mol­ecule lies on an inversion center.

## Related literature

For details of the synthesis and the application of benzothia­zoles, see: Chakraborti *et al.* (2004 ▶); Seijas *et al.* (2007 ▶); Wang *et al.* (2009 ▶). For the use of microwave-assisted organic synthesis, see: Kappe & Stadler (2005 ▶).
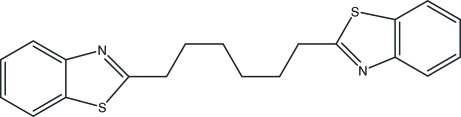


## Experimental

### 

#### Crystal data

C_20_H_20_N_2_S_2_
*M* *_r_* = 342.50
Monoclinic, 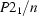

*a* = 5.7590 (12) Å
*b* = 8.3030 (17) Å
*c* = 18.974 (4) Å
β = 96.03 (3)°
*V* = 902.3 (3) Å^3^
*Z* = 2
Mo *K*α radiation
μ = 0.30 mm^−1^
*T* = 293 (2) K
0.30 × 0.20 × 0.10 mm

#### Data collection

Enraf–Nonius CAD-4 diffractometer
Absorption correction: ψ scan (North *et al.*, 1968 ▶) *T*_min_ = 0.916, *T*_max_ = 0.971
1626 measured reflections
1626 independent reflections
1102 reflections with *I* > 2σ(*I*)
3 standard reflections every 200 reflections intensity decay: 9%

#### Refinement

*R*[*F* ^2^ > 2σ(*F* ^2^)] = 0.062
*wR*(*F* ^2^) = 0.182
*S* = 1.01
1626 reflections
109 parameters
H-atom parameters constrained
Δρ_max_ = 0.37 e Å^−3^
Δρ_min_ = −0.40 e Å^−3^

### 

Data collection: *CAD-4 Software* (Enraf–Nonius, 1989 ▶); cell refinement: *CAD-4 Software*; data reduction: *XCAD4* (Harms & Wocadlo, 1995 ▶); program(s) used to solve structure: *SHELXS97* (Sheldrick, 2008 ▶); program(s) used to refine structure: *SHELXL97* (Sheldrick, 2008 ▶); molecular graphics: *SHELXTL* (Sheldrick, 2008 ▶); software used to prepare material for publication: *SHELXTL*.

## Supplementary Material

Crystal structure: contains datablocks global, I. DOI: 10.1107/S1600536809000610/bx2191sup1.cif

Structure factors: contains datablocks I. DOI: 10.1107/S1600536809000610/bx2191Isup2.hkl

Additional supplementary materials:  crystallographic information; 3D view; checkCIF report

## Figures and Tables

**Fig. 1.**
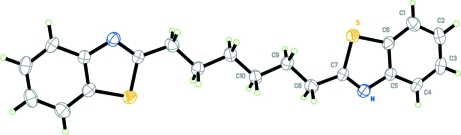
A view of the molecular structure of (I) showing the atom-numbering scheme and 30% displacement ellipsoids. Unlabeled atoms are related to labeled atoms by symmetry code (-x+1, -y, -z).

## supplementary crystallographic information

### Comment

Benzothiazole are remarkable heterocyclic ring systems. They have been found to exhibit a wide spectrum of biological activities. Many kinds of 2-substituted benzothiazoles are utilized as vulcanization accelators in the manufacture of rubber,as fluorescent brightening agents in textile dyeing,and in the leather industry (Chakraborti *et al.*, 2004; Seijas *et al.*, 2007; Wang *et al.*, 2009). There are numerous synthetic methods to produce 2-arylbenzothiazoles. The most important ones include the reaction of *o*-aminothiophenols with benzoic acids or their derivatives (Chakraborti *et al.*, 2004; Seijas *et al.*, 2007; Wang *et al.*, 2009). Microwave-assisted organic synthesis (MAOS) is a powerful technique that is being used more and more to accelerate thermal organic reactions (Kappe & Stadler, 2005). We are focusing on Microwave-assisted synthesis of new products of bisbenzothiazole. We here report the crystal structure of the title compound (I). The atom-numbering scheme of (I) is shown in Fig. 1.The compound lies on an inversion center (symmetry code -x+1, -y, -z ).

### Experimental

A mixture of 2-aminothiophenol (2.5 g, 20 mmol), 5 ml orthophosphoric acid, 5 g polyphosphoric acid and 1,6-hexanedicarboxylic acid (1.74 g, 10 mmol) in a beakerflask (150 ml) was placed in a domestic microwave oven (0.8 KW, 2450 MHz) and irradiated (micromode, full power) for 4 min(30 s per time). The reaction mixture was cooled to r.t. and washed with aq NaOH (20%, 150 ml). The pH was adjusted to 10, the resulted solide was filtered. Then the crude compound (I) was obtained. It was crystallized from ethanol. Crystals of (I) suitable for X-ray diffraction were obtained by slow evaporation of methanol. ^1^H NMR (DMSO, δ, p.p.m.) 7.35–7.40 (m, 2 H), 7.46–7.51 (m, 2 H), 7.64 (dd, 2 H), 7.81 (d, 2 H), 7.95 (dd, 2 H), 8.05 (d, 2 H).

### Refinement

All H atoms were positioned geometrically, with C—H = 0.93 and 0.97 Å for methyl and methylene H atoms, and constrained to ride on their parent atoms, with *U*_iso_(H) = *xU*_eq_(C), where *x*= 1.5 for methyl H and *x* = 1.2 for methylene H atoms.

### Crystal data

**Table:**

C_20_H_20_N_2_S_2_	*F*(000) = 372
*M**_r_* = 342.50	*D*_x_ = 1.297 Mg m^−^^3^
Monoclinic, *P*2_1_/*n*	Mo *K*α radiation, λ = 0.71073 Å
Hall symbol: -P 2yn	Cell parameters from 27 reflections
*a* = 5.7590 (12) Å	θ = 1–25°
*b* = 8.3030 (17) Å	µ = 0.30 mm^−^^1^
*c* = 18.974 (4) Å	*T* = 293 K
β = 96.03 (3)°	Block, yellow
*V* = 902.3 (3) Å^3^	0.30 × 0.20 × 0.10 mm
*Z* = 2	

### Data collection

**Table:**

Enraf–Nonius CAD-4 diffractometer	1102 reflections with *I* > 2σ(*I*)
Radiation source: fine-focus sealed tube	*R*_int_ = 0.0000
graphite	θ_max_ = 25.3°, θ_min_ = 2.2°
ω/2θ scans	*h* = −6→6
Absorption correction: ψ scan (North *et al.*, 1968)	*k* = 0→9
*T*_min_ = 0.916, *T*_max_ = 0.971	*l* = 0→22
1626 measured reflections	3 standard reflections every 200 reflections
1626 independent reflections	intensity decay: 9%

### Refinement

**Table:**

Refinement on *F*^2^	Primary atom site location: structure-invariant direct methods
Least-squares matrix: full	Secondary atom site location: difference Fourier map
*R*[*F*^2^ > 2σ(*F*^2^)] = 0.062	Hydrogen site location: inferred from neighbouring sites
*wR*(*F*^2^) = 0.182	H-atom parameters constrained
*S* = 1.01	*w* = 1/[σ^2^(*F*_o_^2^) + (0.06*P*)^2^ + 1.95*P*] where *P* = (*F*_o_^2^ + 2*F*_c_^2^)/3
1626 reflections	(Δ/σ)_max_ < 0.001
109 parameters	Δρ_max_ = 0.37 e Å^−^^3^
0 restraints	Δρ_min_ = −0.39 e Å^−^^3^

### Special details

**Table:**

Geometry. All e.s.d.'s (except the e.s.d. in the dihedral angle between two l.s. planes) are estimated using the full covariance matrix. The cell e.s.d.'s are taken into account individually in the estimation of e.s.d.'s in distances, angles and torsion angles; correlations between e.s.d.'s in cell parameters are only used when they are defined by crystal symmetry. An approximate (isotropic) treatment of cell e.s.d.'s is used for estimating e.s.d.'s involving l.s. planes.
Refinement. Refinement of *F*^2^ against ALL reflections. The weighted *R*-factor *wR* and goodness of fit *S* are based on *F*^2^, conventional *R*-factors *R* are based on *F*, with *F* set to zero for negative *F*^2^. The threshold expression of *F*^2^ > σ(*F*^2^) is used only for calculating *R*-factors(gt) *etc*. and is not relevant to the choice of reflections for refinement. *R*-factors based on *F*^2^ are statistically about twice as large as those based on *F*, and *R*- factors based on ALL data will be even larger.

### Fractional atomic coordinates and isotropic or equivalent isotropic displacement parameters (Å^2^)

**Table:**

	*x*	*y*	*z*	*U*_iso_*/*U*_eq_	
S	0.21252 (19)	0.52096 (15)	0.08482 (6)	0.0614 (4)	
N	0.6060 (6)	0.5674 (4)	0.16015 (18)	0.0569 (9)	
C1	0.0926 (8)	0.8256 (6)	0.1352 (3)	0.0701 (13)	
H1A	−0.0542	0.8244	0.1096	0.084*	
C2	0.1629 (9)	0.9538 (6)	0.1788 (3)	0.0738 (14)	
H2A	0.0601	1.0387	0.1831	0.089*	
C3	0.3826 (9)	0.9590 (6)	0.2162 (2)	0.0673 (13)	
H3A	0.4266	1.0481	0.2442	0.081*	
C4	0.5351 (8)	0.8342 (5)	0.2123 (2)	0.0573 (11)	
H4A	0.6813	0.8370	0.2382	0.069*	
C5	0.4707 (7)	0.7038 (5)	0.1695 (2)	0.0468 (9)	
C6	0.2468 (7)	0.6990 (5)	0.1308 (2)	0.0539 (10)	
C7	0.4953 (7)	0.4647 (5)	0.1187 (2)	0.0495 (9)	
C8	0.5905 (8)	0.3044 (5)	0.1000 (2)	0.0631 (12)	
H8A	0.7463	0.3214	0.0864	0.076*	
H8B	0.6065	0.2391	0.1426	0.076*	
C9	0.4567 (8)	0.2091 (5)	0.0429 (2)	0.0556 (10)	
H9A	0.4414	0.2725	−0.0002	0.067*	
H9B	0.3009	0.1901	0.0562	0.067*	
C10	0.5656 (8)	0.0488 (5)	0.0278 (2)	0.0583 (11)	
H10A	0.7206	0.0683	0.0139	0.070*	
H10B	0.5836	−0.0134	0.0712	0.070*	

### Atomic displacement parameters (Å^2^)

**Table:**

	*U*^11^	*U*^22^	*U*^33^	*U*^12^	*U*^13^	*U*^23^
S	0.0522 (7)	0.0655 (8)	0.0654 (7)	−0.0031 (5)	0.0018 (5)	−0.0103 (6)
N	0.063 (2)	0.056 (2)	0.051 (2)	−0.0014 (17)	0.0036 (16)	−0.0028 (17)
C1	0.061 (3)	0.075 (3)	0.076 (3)	0.008 (2)	0.018 (2)	0.002 (3)
C2	0.084 (4)	0.065 (3)	0.078 (3)	0.013 (3)	0.035 (3)	−0.007 (3)
C3	0.088 (4)	0.061 (3)	0.058 (3)	−0.011 (3)	0.031 (2)	−0.013 (2)
C4	0.067 (3)	0.063 (3)	0.043 (2)	−0.014 (2)	0.0089 (19)	−0.011 (2)
C5	0.057 (2)	0.044 (2)	0.040 (2)	−0.0024 (17)	0.0089 (17)	0.0032 (17)
C6	0.050 (2)	0.066 (3)	0.047 (2)	−0.006 (2)	0.0127 (18)	−0.006 (2)
C7	0.055 (2)	0.047 (2)	0.046 (2)	−0.0056 (18)	0.0063 (17)	−0.0045 (18)
C8	0.070 (3)	0.053 (3)	0.066 (3)	0.009 (2)	0.005 (2)	0.007 (2)
C9	0.067 (3)	0.052 (2)	0.048 (2)	−0.002 (2)	0.0086 (19)	0.0016 (19)
C10	0.071 (3)	0.053 (2)	0.052 (2)	0.007 (2)	0.012 (2)	0.002 (2)

### Geometric parameters (Å, °)

**Table:**

S—C6	1.717 (4)	C4—H4A	0.9300
S—C7	1.750 (4)	C5—C6	1.416 (5)
N—C7	1.283 (5)	C7—C8	1.496 (6)
N—C5	1.397 (5)	C8—C9	1.489 (6)
C1—C2	1.382 (7)	C8—H8A	0.9700
C1—C6	1.384 (6)	C8—H8B	0.9700
C1—H1A	0.9300	C9—C10	1.512 (6)
C2—C3	1.385 (7)	C9—H9A	0.9700
C2—H2A	0.9300	C9—H9B	0.9700
C3—C4	1.365 (6)	C10—C10^i^	1.473 (8)
C3—H3A	0.9300	C10—H10A	0.9700
C4—C5	1.380 (5)	C10—H10B	0.9700
			
C6—S—C7	89.46 (19)	N—C7—S	115.5 (3)
C7—N—C5	111.6 (4)	C8—C7—S	120.0 (3)
C2—C1—C6	118.1 (5)	C9—C8—C7	118.1 (4)
C2—C1—H1A	120.9	C9—C8—H8A	107.8
C6—C1—H1A	120.9	C7—C8—H8A	107.8
C1—C2—C3	121.7 (5)	C9—C8—H8B	107.8
C1—C2—H2A	119.2	C7—C8—H8B	107.8
C3—C2—H2A	119.2	H8A—C8—H8B	107.1
C4—C3—C2	120.4 (4)	C8—C9—C10	114.4 (4)
C4—C3—H3A	119.8	C8—C9—H9A	108.7
C2—C3—H3A	119.8	C10—C9—H9A	108.7
C3—C4—C5	119.5 (4)	C8—C9—H9B	108.7
C3—C4—H4A	120.2	C10—C9—H9B	108.7
C5—C4—H4A	120.2	H9A—C9—H9B	107.6
C4—C5—N	126.3 (4)	C10^i^—C10—C9	115.4 (5)
C4—C5—C6	120.1 (4)	C10^i^—C10—H10A	108.4
N—C5—C6	113.6 (4)	C9—C10—H10A	108.4
C1—C6—C5	120.1 (4)	C10^i^—C10—H10B	108.4
C1—C6—S	130.1 (4)	C9—C10—H10B	108.4
C5—C6—S	109.7 (3)	H10A—C10—H10B	107.5
N—C7—C8	124.4 (4)		
			
C6—C1—C2—C3	1.3 (7)	N—C5—C6—S	0.9 (4)
C1—C2—C3—C4	−1.6 (7)	C7—S—C6—C1	−179.0 (5)
C2—C3—C4—C5	1.3 (7)	C7—S—C6—C5	−0.5 (3)
C3—C4—C5—N	−179.7 (4)	C5—N—C7—C8	−178.0 (4)
C3—C4—C5—C6	−0.8 (6)	C5—N—C7—S	0.5 (5)
C7—N—C5—C4	178.0 (4)	C6—S—C7—N	0.0 (3)
C7—N—C5—C6	−0.9 (5)	C6—S—C7—C8	178.6 (4)
C2—C1—C6—C5	−0.8 (7)	N—C7—C8—C9	−170.9 (4)
C2—C1—C6—S	177.5 (4)	S—C7—C8—C9	10.6 (6)
C4—C5—C6—C1	0.5 (6)	C7—C8—C9—C10	−179.9 (4)
N—C5—C6—C1	179.6 (4)	C8—C9—C10—C10^i^	179.1 (5)
C4—C5—C6—S	−178.1 (3)		

Symmetry codes: (i) −*x*+1, −*y*, −*z*.
